# The Australian Youth Cancer Service: Developing and Monitoring the Activity of Nationally Coordinated Adolescent and Young Adult Cancer Care

**DOI:** 10.3390/cancers13112675

**Published:** 2021-05-28

**Authors:** Pandora Patterson, Kimberley R. Allison, Helen Bibby, Kate Thompson, Jeremy Lewin, Taia Briggs, Rick Walker, Michael Osborn, Meg Plaster, Allan Hayward, Roslyn Henney, Shannyn George, Dominic Keuskamp, Antoinette Anazodo

**Affiliations:** 1 Research, Evaluation and Policy Unit, CanTeen, Sydney, NSW 2042, Australia; kimberley.allison@canteen.org.au (K.R.A.); helen.bibby@canteen.org.au (H.B.); dominic@anzdata.org.au (D.K.); 2 Faculty of Medicine and Health, University of Sydney, Sydney, NSW 2006, Australia; 3 Victoria/Tasmania Youth Cancer Service, Peter MacCallum Cancer Centre, Melbourne, VIC 3000, Australia; kate.thompson@petermac.org (K.T.); jeremy.lewin@petermac.org (J.L.); 4 ONTrac at PeterMac Victorian Adolescent and Young Adult Cancer Service, Peter MacCallum Cancer Centre, Melbourne, VIC 3000, Australia; 5 Faculty of Medicine, Dentistry and Health Sciences, University of Melbourne, Melbourne, VIC 3010, Australia; 6 Department of Cancer Medicine, Peter MacCallum Cancer Centre, Melbourne, VIC 3000, Australia; 7 Sir Peter MacCallum Department of Oncology, University of Melbourne, Melbourne, VIC 3010, Australia; 8 New South Wales/Australian Capital Territory Youth Cancer Service, Sydney, NSW 2031, Australia; taia.briggs@health.nsw.gov.au (T.B.); antoinette.anazodo@health.nsw.gov.au (A.A.); 9 Nelune Comprehensive Cancer Centre, Prince of Wales Hospital, Sydney, NSW 2031, Australia; 10 Queensland Youth Cancer Service, Queensland Children’s Hospital, Brisbane, QLD 4101, Australia; rick.walker@health.qld.gov.au (R.W.); roslyn.henney@health.qld.gov.au (R.H.); 11 Oncology Services Group, Children’s Health Queensland, Brisbane, QLD 4000, Australia; 12 Princess Alexandra Hospital, Brisbane, QLD 4102, Australia; 13 School of Medicine, University of Queensland, Brisbane, QLD 4072, Australia; 14 South Australia/Northern Territory Youth Cancer Service, Royal Adelaide Hospital, Adelaide, SA 5000, Australia; michael.osborn@health.sa.gov.au (M.O.); allan.hayward@health.sa.gov.au (A.H.); 15 Department of Haematology and Oncology, Women’s and Children’s Hospital, Adelaide, SA 5006, Australia; 16 Western Australia Youth Cancer Service, Sir Charles Gairdner Hospital, Nedlands, WA 6009, Australia; megan.plaster@health.wa.gov.au (M.P.); shannyn.george@health.wa.gov.au (S.G.); 17 Sir Charles Gairdner Hospital, Nedlands, WA 6009, Australia; 18 School of Women and Children’s Health, Faculty of Medicine, University of New South Wales, Sydney, NSW 2052, Australia; 19 Kids Cancer Centre, Sydney Children’s Hospital, Sydney, NSW 2031, Australia

**Keywords:** activity data, adolescent and young adult, clinical trial participation, oncofertility, psychosocial, service delivery, survivorship

## Abstract

**Simple Summary:**

A cancer diagnosis during adolescence or young adulthood presents unique medical and psychosocial challenges which must be addressed in the provision of quality, comprehensive cancer care. Tailoring services to the needs of this population requires careful work to identify, monitor and evaluate areas of care; however, published work in this area to guide service priorities is limited. This paper presents work done by the Australian Youth Cancer Services to operationalise and deliver quality care to adolescents and young adults with cancer, focusing on nationally coordinated service improvement initiatives and activity data collection in four areas that are of particular concern to young people diagnosed with cancer: clinical trial enrolment, oncofertility, psychosocial care and survivorship. This account may be instructive for health services seeking to improve the delivery and monitoring of cancer care provided to adolescents and young adults.

**Abstract:**

Adolescents and young adults (aged 15–25 years) diagnosed with cancer have unique medical and psychosocial experiences and care needs, distinct from those of paediatric and older adult patients. Since 2011, the Australian Youth Cancer Services have provided developmentally appropriate, multidisciplinary and comprehensive care to these young patients, facilitated by national service coordination and activity data collection and monitoring. This paper reports on how the Youth Cancer Services have conceptualised and delivered quality youth cancer care in four priority areas: clinical trial participation, oncofertility, psychosocial care and survivorship. National activity data collected by the Youth Cancer Services between 2016–17 and 2019–20 are used to illustrate how service monitoring processes have facilitated improvements in coordination and accountability across multiple indicators of quality youth cancer care, including clinical trial participation, access to fertility information and preservation, psychosocial screening and care and the transition from active treatment to survivorship. Accounts of both service delivery and monitoring and evaluation processes within the Australian Youth Cancer Services provide an exemplar of how coordinated initiatives may be employed to deliver, monitor and improve quality cancer care for adolescents and young adults.

## 1. Introduction

Cancer in adolescents and young adults (AYAs; defined as 15–25 years in Australia) is relatively rare, with approximately 1200 young Australians newly diagnosed each year [1]. A diagnosis at this age presents unique issues, including prolonged pathways to diagnosis, treatment across paediatric and adult settings, disrupted developmental transitions and educational, vocational, social and relational challenges [2,3,4]. These distinctive features necessitate tailoring services to the unique needs of the AYA population to provide quality, developmentally appropriate cancer care. This paper presents an account of how quality youth cancer care is conceptualised, monitored and improved in the Australian setting, using activity data collected from all state/territory youth cancer services to illustrate service evaluation processes; we focus on four priority areas for AYA cancer care (clinical trial enrolment, oncofertility, psychosocial care and survivorship). This is the most comprehensive dataset on Australian youth cancer care to date, covering details of the care received by all AYAs across the nation who have accessed support through the Youth Cancer Services between 2016 and 2020.

## 2. A National Approach to Improving AYA Cancer Care: The Australian Youth Cancer Service

Australia has a dual public/private healthcare system, with all citizens and permanent residents able to access universal healthcare (including public hospital services and subsidised medications) through the tax-funded Medicare scheme. Private health insurance offers alternative options for treatment (e.g., through private hospitals) and non-medical health services; however, the majority of AYAs with cancer receive specialist care through the public hospital system [5]. This is complemented by additional services (e.g., information, counselling, peer support, advocacy) provided by community and not-for-profit organisations.

Recognising the unique challenges faced by AYAs, in 2008, Cancer Australia and Canteen developed the National Service Delivery Framework (NSDF), which articulated a strategic direction for the provision of specialist, multidisciplinary AYA cancer care [6] (Canteen is the Australian organisation for adolescents and young adults (12–25 yrs) who have been impacted by their own or a family member’s cancer diagnosis. Canteen has youth-specific treatment teams who provide evidence-based support through information provision, individual case management, counselling and therapeutic programs (www.canteen.org.au).). Elements identified as key components of quality care for this population include national coordination of cancer services, increased access to support services and clinical trials, comprehensive medical and psychosocial assessment, coordinated care and multidisciplinary expertise in AYA care [6]. This framework guided the subsequent establishment of the Youth Cancer Service (YCS), which was launched in 2010, and, in 2017, the Australian Youth Cancer Framework (AYCF; [7]) was developed to progress the national vision for AYA cancer care, “ensuring every young person with cancer has access to best-practice, age-appropriate care and support for their survival, health, and meaningful participation across all areas of life”. 

Funded by Federal and State governments, with co-investment and management by Canteen, the YCS provides age-appropriate medical, psychosocial and practical care to AYAs across Australia who require hospital-based treatment for their cancer [8]. The number of AYAs receiving care through the YCS has increased steadily in recent years, reaching 1851 in 2019–20 (Table 1). Patients are predominantly from metropolitan areas near treatment centres (~70%), although a significant minority are from regional/rural areas (15–25%) or travel interstate or internationally to receive care (3–8%)—approximately reflecting the distribution of the Australian population [9]. Around 40% of patients are new presentations to the YCS, primarily those whose cancers have been newly diagnosed. The YCS now supports approximately three quarters of AYAs who require hospital-based cancer treatment nationally. 

The YCS comprises five state/territory teams based in major hospitals, which work collaboratively with other hospitals and clinicians across the country according to local geographic and demographic needs (Table 2) [5]. Several features are common across each state/territory, including collaboration across paediatric and adult sectors (with many clinicians dually trained in both paediatric oncology and adult oncology or haematology, or having undertaken AYA oncology fellowships), care coordination and integrated multidisciplinary care [5,6,7]. Key amongst these is the collaborative approach to care: the integration of each YCS within acute health services allows patients to benefit from both the age-specific expertise of YCS staff and the tumour-stream expertise of local cancer teams, resulting in tailored care that is disease-specific and developmentally appropriate. This is reflected in the regular multidisciplinary team meetings in each jurisdiction, at which the care of each YCS patient is discussed (e.g., [10]), as well as the attendance of YCS clinicians in tumour-stream meetings concerning the care of AYA patients. This approach to collaborative care is supported by clinical leadership within each jurisdiction: the designation of a lead clinician for each jurisdiction allows focused efforts to build clinical networks with paediatric and adult-treating clinicians; service managers coordinate local and national approaches to service delivery, including integration with partnered services; and the assignment of care coordinators for each patient provides support between YCS and local services and facilitates seamless, comprehensive cancer care. YCS teams are completed by medical, nursing, allied health professionals and support personnel. Connections between jurisdictions further facilitate service delivery alignment to ensure that all services are run consistently with the YCS strategic plan, NSDF and AYCF; these connections further improve access to clinical care and trials between states and allow for mentorship and peer support between YCS clinicians.

Critically, the integrated approach of the YCS allows for coordinated national efforts regarding service improvement, professional development, networking, research and consumer engagement, as well as national collection of activity data to monitor service performance in areas identified as priorities by the NSDF and AYCF [6,7]. 

## 3. Service Monitoring and Improvement in the Australian Youth Cancer Service

Collecting and monitoring activity data is an essential component in assessing the performance of healthcare systems and driving and evaluating the impact of service improvement initiatives [13,14,15]. This is just one element of service evaluation and improvement within the YCS and is complemented by data linkage projects and evaluation of patients’ experiences of care.

The five YCS jurisdictions provide quarterly data on service provision to Canteen for national aggregation, and these aggregated annual data from 2016–17 to 2019–20 are reported and discussed here (activity data for survivorship care were only collected from 2017–18 onwards. The final year of this reporting period overlapped with the initial months of the COVID-19 pandemic; however, the impact of the pandemic was relatively minimal in Australia compared to other countries, and there was limited change in YCS activity during this time.). As data presented are aggregated count data rather than individual patient data, no statistical analysis to determine the statistical significance of change over time is possible. Rather, we present the data to illustrate their utility for service monitoring and improvement.

Participation in activity data collection is a requirement of the YCS contract and is overseen in each jurisdiction by a nominated staff member (typically a research nurse). These data are typically collated from existing hospital electronic records and custom processes; the precise means of doing so therefore varies between YCS jurisdictions, in line with local data collection requirements and available information systems. Indicators were chosen for their importance as key components of quality AYA cancer care, per previous research and clinical stakeholder consultation; while these do not fully capture the breadth of services provided, it was necessary to limit the number of indicators collected to those identified as priorities in order to reduce the burden of reporting for YCS staff (as this necessitated collation and de-duplication of data from multiple hospital sites and electronic medical record platforms) [11]. Activity data items were operationalised nationally; however, there is some variability in how individual states/territories interpreted variables in accordance with local procedural requirements. Following the reporting and aggregation of activity data each quarter, a teleconference is held with each state/territory YCS to reflect on and discuss figures which diverge from previously set targets (where relevant) or activity trends and identify potential contributing factors and means of improving service provision in these areas in the future.

The process of service development, coordination, monitoring and ongoing improvement can be demonstrated by examining four key aspects of AYA cancer care: clinical trial participation; oncofertility care; psychosocial assessment and care; and the transition from treatment to survivorship care [6,7]. (Activity data are also collected on other aspects of care, including secondary consultations, coordination of care, referral to community-based services, and participation in research other than medical clinical trials; however, these indicators are not the focus of this paper.)

## 4. Enrolment in Clinical Trials

Participation in medical clinical trials is associated with improved outcomes [16,17], yet enrolment in trials is lower amongst AYAs and survival outcomes for certain cancers have not improved to the same extent as for other age groups [18,19,20]. In Australia, key barriers to AYA enrolment in clinical trials begin at the system level: the low number and geographical dispersion of AYAs with cancer (compounded by age restrictions on paediatric and adult sites) means that individual services rarely meet the number of enrolments required to participate in international trials [5], contributing to poor trial availability in Australian services [18]. YCS initiatives to improve medical clinical trial enrolment have thus focused on increasing trial availability through advocacy for improved coordination, investment and infrastructure (e.g. [18]) and through Canteen securing funding from the Australian Government Medical Research Future Fund for the Australian Young Cancer Patient Clinical Trials Initiative [21]. This is complemented by efforts to improve clinicians’ awareness of and access to information about available AYA trials, including the development of the AYA (15–25 years olds) cancer trials app to facilitate the identification of trials appropriate to patients in this age group [22].

**Figure 1:**
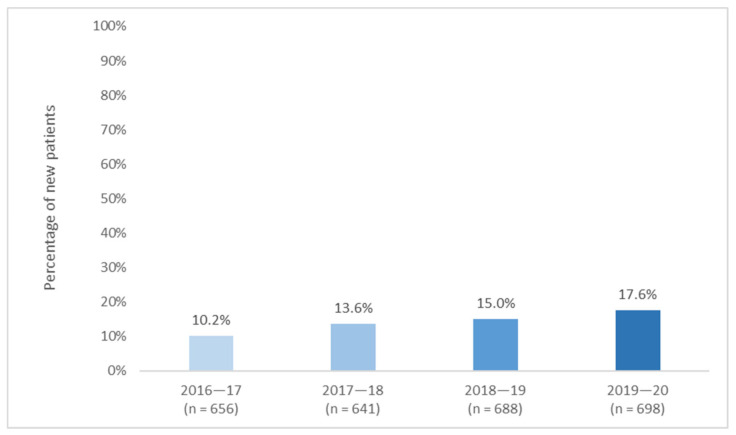
YCS activity data on medical clinical trial enrolment, 2016–17 to 2019–20.

The number of AYAs enrolled in medical clinical trials (any phase) increased from 67 in 2016–17 to 123 in 2019–20, representing 10-18% of new YCS patients (Figure 1; data available in Supplementary Table S1). Notably, this may be an underestimate as AYAs who are referred to a clinical trial through a non-YCS clinician (e.g., if their medical care is managed by a local specialist) may not be included in these counts. Additionally, these figures indicate the proportion of YCS patients who participated in a clinical trial in that year, rather than the proportion who participated at any point during their cancer experience, as is typically reported, complicating comparisons between studies. However, these numbers are considerably higher than previously reported enrolment figures in some United States studies (5.5–7.6%; [23,24,25]), and comparable to others (13–18%; [26,27,28,29]), although they lag behind the UK, where coordinated, multifaceted initiatives have helped to increase participation rates to 20.6% [30]. National comparison data are not available for other age groups in Australia [31,32] but YCS trial enrolment rates also exceed those reported for adults in NSW and Victoria (7–9%; [33,34]), although they fall short of paediatric rates (>50%; [35]), which is likely attributable to the embedding of trials into standard care [18]. While the apparent recent increase in AYA trial participation is encouraging, further efforts to improve enrolment rates are needed, particularly in light of the low numbers and geographical dispersion of Australian AYAs [18]. For example, telemedicine has facilitated the remote participation of regional patients [36] and may enable AYA participation in larger international trials or at sites that their age may restrict them from accessing. Collective advocacy by Canteen and the YCS may also help to secure further funding for AYA-specific clinical trials. Similar efforts may also help to improve participation in other forms of research, including translational studies and clinical trials of psychosocial interventions, which are also critical in improving survivorship outcomes.

## 5. Oncofertility Care

The risk of infertility is identified by cancer patients as one of the top unmet needs in survivorship [37,38], resulting in fertility-related distress, including increased risks of depression, anxiety and grief, and contributes to reduced relationship satisfaction and quality of life [39,40,41,42,43,44,45,46,47]. Despite this, there is often limited knowledge and discussion of cancer-related fertility issues across the cancer trajectory, and little awareness of and access to fertility preservation services and reproductive survivorship care [43,48,49,50,51,52,53]. In identifying oncofertility as a priority area for the YCS, the NSDF and AYCF specified that quality care would result in AYAs being aware of potential impacts of treatment on fertility, and of fertility preservation options [6,7]. At the service level, YCS initiatives to improve the rate and quality of fertility discussions have included professional development and communication training, resource development, awareness-raising events, introduction of patient peer support and research (e.g. [54,55]). Canteen’s collaboration with the Clinical Oncology Society of Australia has also led to the development of evidence-based guidance and recommendations regarding the discussion of treatment impacts on fertility and preservation options [56,57], while collaboration with the Future Fertility team has resulted in joint advocacy for the subsidisation of oncofertility care (Future Fertility is a research group that was set up in 2013 to conduct preclinical and clinical studies that cover oncofertility care from diagnosis to survivorship (www.futurefertility.com.au). 

**Figure 2:**
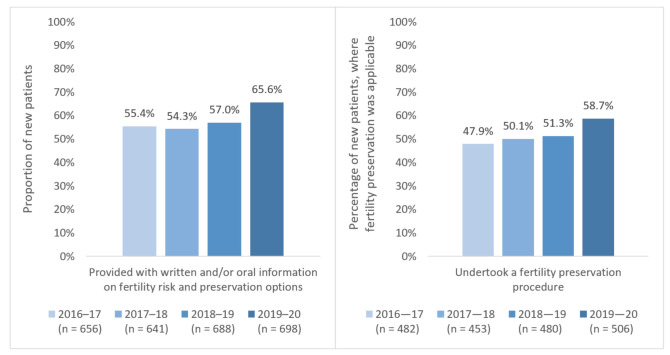
YCS activity data on oncofertility care, 2016–17 to 2019–20.

Following a series of education and awareness initiatives and resource development in 2015–16, combined with jurisdiction-specific work to implement models of care and referral pathways, the proportion of newly diagnosed YCS patients receiving information about fertility risk and preservation options rose sharply [58]. In the subsequent years, these rates increased further from 54–55% to 66% in 2019–20 (Figure 2), well above published international rates [59]. These figures are encouraging, indicating that almost two thirds of newly diagnosed AYAs are receiving information which may better inform their decision making, enable timely access to fertility preservation and allow early detection and intervention for fertility distress [45,60]—and, indeed, the proportion of new patients undertaking fertility preservation procedures (defined as any procedure aiming to save or protect eggs, embryos, sperm or reproductive tissue, e.g. egg harvesting, embryo freezing, sperm banking, zolodex) also increased during this period. However, they also indicate a substantial proportion of AYAs who were not informed about fertility preservation despite the strategies implemented, and reasons for this must be further examined. One significant barrier is high fertility preservation and storage costs, which may lead to clinicians’ hesitancy to refer patients based on income, perceived need for treatment, disease prognosis and/or predicted success of procedures. Advocacy by Canteen, YCS, the Future Fertility team and the Medical Oncology Group of Australia has helped to secure subsidies for some preservation options (sperm banking, ovarian transposition, zoladex) as well as reduced-price or free services at some fertility centres [59,61], improving equity of access to oncofertility care. Ongoing work will include further developing referral pathways to improve timely access to fertility consultations and expertise (who may provide more individualised risk assessments) [62]; regular review and updating of oncofertility resources; and training all staff (including regular onboarding of new staff) to support and deliver oncofertility care. Our efforts focus on YCS centres and clinicians, but prioritisation of oncofertility care in national cancer plans and clinician training will help to extend this to AYAs treated elsewhere.

## 6. Psychosocial Care

The intersection between AYAs’ dynamic developmental stage and the disruption associated with cancer creates unique psychosocial challenges [63,64], contributing to higher levels of psychological symptomatology [65,66] and supportive care needs [67,68], and poorer quality of life, coping and health outcomes [69,70,71,72]. Routine, regular and repeated screening and assessment of psychosocial issues and needs across the cancer trajectory allows for the timely identification of these concerns and the adjustment of care in response to changing needs, and it guides the provision of responsive and efficient services [63,64,73], but this depends on the availability of developmentally appropriate instruments [63,64,74]. Accordingly, the NSDF identified the development of age-specific psychosocial assessment tools and processes as a key priority for care in the YCS [6], leading to the development of the AYA Oncology Psychosocial Care Manual (available via https://www.canteen.org.au/youth-cancer/resources/aya-psychosocial-care-manual/) [63,75] and AYA Psychosocial Oncology Screening Tool (the AYA-POST; [73]). The manual details the psychosocial care pathway (screening, care planning and assessment) implemented within the YCS, ensuring that psychosocial concerns are identified and addressed in a timely and comprehensive way [63] as part of the national effort to improve young patients’ access to targeted and responsive psychosocial care [6,7,76]. A key performance indicator (KPI; reportable to the Federal Department of Health) of 75% of newly diagnosed patients in 2017–18 and 2018–19 completing the AYA-POST was also set, with the intention of further galvanising action and driving service improvement [13,14].

**Figure 3:**
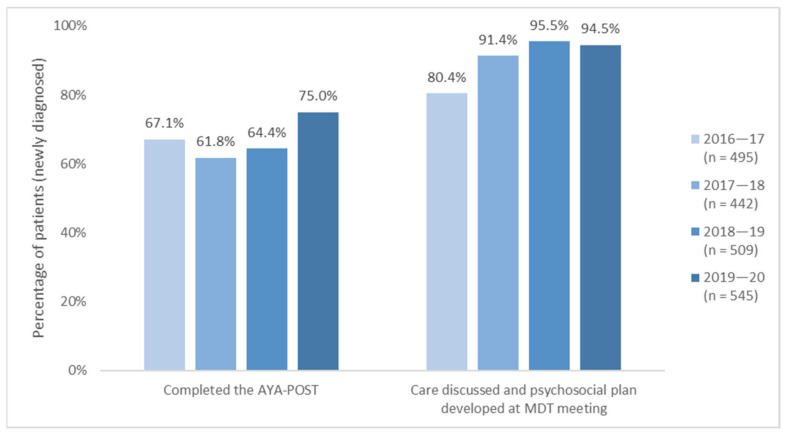
YCS activity data on psychosocial care, 2016–17 to 2019–20.

Between 2016–17 and 2018–19, the proportion of newly diagnosed YCS patients completing the AYA-POST averaged 64%, peaking at 67.1% in 2016–17 but falling short of the KPI target of 75% (Figure 3). An independent YCS evaluation report in 2020 noted that contributing factors included: “some clinicians administer a single comprehensive assessment tool rather than the use of a screening tool as part of a stepped care assessment approach (initial screening, followed by more comprehensive assessment as required); (2) patients being counted as new referrals in a reporting period but not screened until the following reporting period; and (3) psychosocial staffing shortages” ([11] p. 18). For the 2019–20 period, however, the rate of AYA-POSTs completed rose to meet the KPI of 75%. It is important to note that psychosocial screening and assessment will only benefit patients if services respond promptly and appropriately to the needs identified [77], and this was the case with the YCS: nearly all newly diagnosed AYAs had their care discussed and a psychosocial care plan developed at a multidisciplinary team meeting, with this proportion increasing from 80% in 2016–17 to approximately 95% in 2018–19 and 2019–20. Incorporating these practices as standards of care helps to establish psychosocial issues as a priority and addresses previously reported systemic barriers to accessing support around poor awareness, availability and integration of services across contexts [78]. By systematically screening, identifying and planning to address AYAs’ psychosocial concerns as part of regular care, clinicians may tailor care to patients’ specific needs, potentially preventing their escalation into more persistent or serious problems [63].

## 7. Survivorship Care

An increasing number of AYAs are being diagnosed with cancer, completing curative treatment and moving into survivorship [79], with significant long-term and late effects [38]. The transition into longer-term survivorship can be challenging, particularly as social and professional support is often lacking [38,80,81,82], leaving AYAs ill-equipped to manage their follow-up care [67,81,83,84]. These factors suggest a need for improved information provision, routine assessment of support needs and identification of appropriate support services by treatment completion. Australian initiatives to improve survivorship care include research and stakeholder consultation to inform service design and delivery [38,85] and the development of an AYA Oncology Psychosocial Survivorship Care Manual (available via https://www.canteen.org.au/youth-cancer/resources/aya-oncology-psychosocial-survivorship-care-manual/) [86] to parallel the AYA Psychosocial Care Manual for AYAs undergoing treatment. This complements state/territory survivorship initiatives tailored to jurisdictional needs and governance requirements, including workshops, survivorship and late effects clinics and resource development [85]. Developing partnerships with community organisations offers additional referral options for AYA survivors seeking specific forms of support and aids in the transition to community-based care. In 2016, target KPIs were set for three aspects of YCS survivorship care: the completion of psychosocial survivorship assessments, care plans and referral to community-based services (with targets for each indicator set at 45% of YCS patients completing treatment in 2017–18 and 55% in 2018–19).

**Figure 4:**
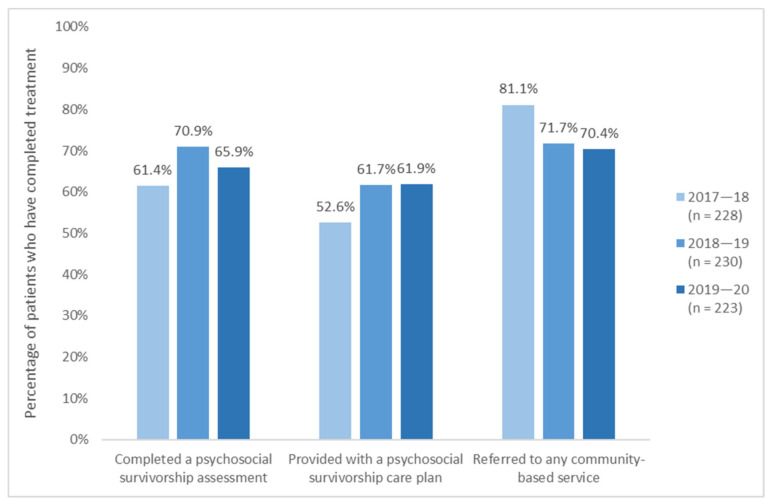
YCS activity data on survivorship care, 2016–17 to 2019–2020.

Of those AYAs who completed active medical treatment in 2017–18, 61% completed a psychosocial survivorship assessment and 53% were provided with a psychosocial survivorship care plan; psychosocial survivorship assessment rates rose to 71% in 2018–19 before declining slightly in 2019–20, while rates of survivorship care plan rose and stabilised around 62% (Figure 4). The provision of psychosocial survivorship care plans has rarely been discussed in the previous literature; however, these rates far exceed those reported in studies of medical survivorship care plans. For example, studies of AYA patients have found that between 0% [38] and 30% [84] reported receiving a medical survivorship care plan, and Birken and Mayer [87] likewise estimated implementation rates at 12–43%. This performance is likely a product of the YCS’ emphasis on comprehensive, multidisciplinary care, reflected in investment into hiring psychosocial staff and developing standardised screening and assessment procedures [6,7,86]. Additionally, 70–80% of survivors were referred to community-based services prior to treatment completion, facilitated by strong partnerships between the YCS and various community-based services. Referrals are particularly important for survivors, as subsequent reductions in AYA–clinician contact can make locating and accessing support services challenging [38,80]. On all measures of survivorship care, the YCS surpassed KPI targets (45% in 2017–18 and 55% in 2018–19, for each indicator). 

## 8. Conclusions

Quality cancer care for AYAs is necessarily complex and multifaceted: the intersection of developmental needs with impacts of cancer and treatment creates unique challenges, which must be addressed if care is to be truly comprehensive. This paper demonstrates how the YCS facilitates Australian AYAs’ access to quality care that is comprehensive and age-appropriate—particularly around access to clinical trials, oncofertility care, psychosocial assessment and support and survivorship care. 

This work may be illustrative for service providers and health professionals seeking to monitor and improve care provision for AYAs with cancer using activity data. It should be noted that aggregated activity data collected primarily for monitoring purposes cannot be used to evaluate whether changes in indicators over time are statistically significant or attributable to specific service improvement initiatives, nor do they capture patients’ subjective experiences of care. However, these data are crucial in monitoring and identifying areas of service provision for further improvement: they capture how care is being provided across the entire patient population, allowing more accurate reporting of the proportion of AYAs engaged with specific aspects of care (e.g., clinical trial enrolment, psychosocial assessment), and are collected and reviewed regularly enough to allow for timely implementation and adjustment of service improvement initiatives [13,14,15]. In the Australian YCS, this has facilitated ongoing work to provide best-practice, comprehensive care to AYAs diagnosed with cancer, as part of a suite of ongoing service evaluation and improvement work—for example, a series of interventions which aimed to improve the provision of oncofertility care [54]. A YCS initiative is underway to establish a national AYA oncology dataset, which will enable more comprehensive collection and linkage of data on service delivery and better facilitate service planning and improvement. A study assessing patient-reported experiences of care within the YCS is also in progress; this is an important component of service evaluation, allowing for analysis of the equity and impacts of access to quality cancer care, providing further direction as to how the YCS can be improved. 

Australia is not entirely unique in its approach to AYA cancer care, with other countries deploying specialised AYA programs with varying degrees of cross-institutional coordination [12]. Where coordinated networks of AYA-specific cancer centres exist, the approach adopted by the Australian YCS may be illustrative in demonstrating how concerted cross-institutional initiatives can be employed to drive improvements in cancer care for young people, and in how connections between paediatric and adult services and clinicians can be leveraged to provide integrated AYA cancer care. Further, all institutions providing care to AYAs with cancer may find the YCS’ approach to service development and evaluation a useful model for their own practice, particularly with respect to the use of activity data to monitor and adapt service provision.

## Figures and Tables

**Table 1:** Numbers of patients supported through the YCS, 2016–17 to 2019–2020.

	2016–17	2017–18	2018–19	2019–20
Total number of patients supported through the YCS	1539	1647	1759	1851
Total number of new patients	656	641	688	698
New patients by cancer situation				
New diagnosis	495	442	509	545
Relapse	58	68	64	50
Other ^a^	103	131	115	103
New patients by residence ^b^				
Metropolitan	476	445	473	477
Regional/rural	101	144	177	187
Interstate/international	21	51	30	31
Number of patients completing medical treatment ^c^	-	228	230	223

^a^ Includes AYAs referred after diagnosis (e.g., those who began treatment in a different setting, or those who were diagnosed before 15 years and ‘aged into’ the YCS) ^b^ Missing data from some patients mean that the sum across areas of residence is less than the total number of new patients ^c^ Data not collected in 2016–17; numbers likely to be an underestimate as some patients received only psychosocial care from the YCS.

**Table 2:** YCS service descriptions.

YCS Jurisdiction	Service Description
New South Wales/Australian Capital Territory	YCS hubs based at two joint adult–paediatric and one adult hospital, with multidisciplinary teams at each. YCS staff also based at four additional partner hospitals (both adult and paediatric) [11]
Queensland	Network linking five tertiary adult and paediatric services through formal partnerships and agreements. Central team based at paediatric treatment centre, with YCS clinicians also located across partner sites—medical management largely managed by local adult or paediatric teams [10]
South Australia/Northern Territory	Centralised YCS team moving between adult and paediatric hospitals, with clinicians also visiting other hospitals. Clinicians both provide primary treatment and secondary consultations to other services [5,12]
Victoria/Tasmania	Centralised service located in a comprehensive cancer centre, with partner sites supporting other providers of AYA cancer care through secondary consultations and workforce capacity building initiatives [5]
Western Australia	Centralised YCS team based at adult hospital cancer centre, with clinicians providing outreach services to public and private treating sites in metropolitan area and telehealth outreach to patients and teams in regional areas [5]

## Data Availability

The data presented in this study are available in this article and supplementary material.

## Supplementary Materials

The following are available online at https://www.mdpi.com/article/10.3390/cancers13112675/s1, Supplementary Table S1: YCS activity data on clinical trial enrolment, oncofertility care, psychosocial care, and survivorship care, 2016–17 to 2019–20.
