# Supplementary material for: The Australian Youth Cancer Service: Developing and Monitoring the Activity of Nationally Coordinated Adolescent and Young Adult Cancer Care
Source: Cancers (Basel). 2021 May 28;13(11):2675. doi: 10.3390/cancers13112675 (PMC8198716; doi:10.3390/cancers13112675)
Supplement: Supplementary file 1 [file cancers-13-02675-s001.zip › cancers-1211040-supplementary.pdf]

Supplementary Table S1

YCS activity data on clinical trial enrolment, oncofertility care, psychosocial care, and survivorship care, 2016-17 to 2019-20

	2016-17	2017-18	2018-19	2019-20
**Enrolment in clinical trials**				
Number/percentage of patients (new):				
Newly enrolled in medical clinical trials	67 (10.2%)	87 (13.6%)	103 (15.0%)	123 (17.6%)
**Oncofertility care**				
Number/percentage of patients (new):				
Provided with written and/or oral information on fertility risk and preservation options	364 (55.4%)	348 (54.3%)	392 (57.0%)	458 (65.6%)
Where fertility preservation was applicable[a]	482 (73.5%)	453 (70.7%)	480 (69.8%)	506 (72.5%)
Number/percentage of patients (new, where fertility preservation was applicable), who:				
Undertook a fertility preservation procedure	231 (47.9%)	227 (50.1%)	246 (51.3%)	297 (58.7%)
**Psychosocial care**				
Number of patients (newly diagnosed) who:				
Completed the AYA-POST	332 (67.1%)	273 (61.8%)	328 (64.4%)	409 (75.0%)
Had their care discussed and psychosocial care plan developed at an MDT meeting	398 (80.4%)	404 (91.4%)	486 (95.5%)	515 (94.5%)

**Survivorship care**[b]				
Number/percentage of patients (who completed treatment):				
Who completed a psychosocial survivorship assessment	-	140 (61.4%)	163 (70.9%)	147 (65.9%)
Who were provided with a survivorship care plan	-	120 (52.6%)	142 (61.7%)	138 (61.9%)
Who were referred to any community-based service	-	185 (81.1%)	165 (71.7%)	157 (70.4%)

[a]Fertility preservation may not be applicable to patients if, for example, treatment is unlikely to impact their fertility.

[b]These indicators were not collected until 2017-18
